# Sweet beverages and the risk of colorectal cancer: the Norwegian Women and Cancer Study

**DOI:** 10.1186/s12885-025-13835-4

**Published:** 2025-04-01

**Authors:** Marie Hauan, Charlotta Rylander, Guri Skeie

**Affiliations:** 1 Department of Community Medicine, Faculty of Health Sciences, UiT The Arctic University of Norway, Tromsø, Norway; 2 IARC Nutrition and Metabolism Branch, International Agency for Research on Cancer, Lyon, France

**Keywords:** Sugar-sweetened beverages, Artificially sweetened beverages, Fruit juices, Diet, Colorectal cancer risk, Colorectal neoplasms, Female, The NOWAC study, Prospective studies, Repeated measures

## Abstract

**Background:**

Colorectal cancer (CRC) is the third most common type of cancer worldwide, with Norwegian women having the highest incidence rate of colon cancer in 2022. The consumption of sweet beverages is a suggested modifiable risk factor for CRC; however, current evidence is limited and inconclusive.

**Objective:**

To assess the associations between the intake of sugar-sweetened beverages (SSBs), artificially sweetened beverages (ASBs), and juice and the risk of overall and subsite-specific CRC among Norwegian women.

**Methods:**

In this prospective cohort study, we included 73,921 participants aged 41–61 years at baseline. Information on sweet beverage consumption was collected using self-reported food frequency questionnaires at two time points between 1998 and 2014. We used Cox proportional hazards models to estimate hazard ratios (HRs) with corresponding 95% confidence intervals (CIs) for the associations between sweet beverage consumption and the risk of overall CRC, proximal colon cancer, distal colon cancer, and rectal cancer.

**Results:**

During a mean follow-up time of 16.5 years from baseline, 1,187 women were diagnosed with CRC. Compared to no consumption, juice consumption was inversely associated with overall CRC risk (HR_≥ 7 glasses/wk_ = 0.81, 95% CI: 0.67–0.98; *p*-trend = 0.025), colon cancer (HR_≥ 7 glasses/wk_ = 0.73, 95% CI: 0.58–0.94; *p*-trend = 0.015) and proximal colon cancer (HR_≥ 7 glasses/wk_ = 0.71, 95% CI: 0.52–0.99; *p*-trend = 0.065) after adjusting for age, education, and diabetes status at baseline. No associations were observed between juice consumption and distal colon cancer or rectal cancer risk, or between the intake of SSBs or ASBs and CRC.

**Conclusion:**

We observed no substantial association between the intake of SSBs or ASBs and the risk of CRC or cancer in colorectal subsites in our cohort of Norwegian women. Conversely, our results indicate that juice consumption is associated with a reduced risk of CRC, particularly in the colon. These results warrant further investigation in larger cohorts with power to detect possible differences in cancer risk across colorectal subsites, especially as patterns of sweet beverage consumption are changing.

**Supplementary Information:**

The online version contains supplementary material available at 10.1186/s12885-025-13835-4.

## Introduction

The incidence rate of colorectal cancer (CRC) has declined over the past decade in Norway [1]. However, 4,912 cases were diagnosed in 2023, and Norwegian women had the highest incidence rate of colon cancer worldwide in 2022 [1, 2]. Globally, CRC was the third most frequently diagnosed cancer and the second leading cause of cancer-related deaths in 2022 [2]. Although incidence rates are declining in many countries with high Human Development Indexes (HDIs), the rates are still the highest in high HDI areas. In contrast, the low/medium HDI areas have increasing incidence rates. These regional differences are attributed to lifestyle changes and access to cancer screening and treatment [2, 3].

Most cases of CRC are adenocarcinomas and of sporadic origin [3]. Further, cumulative evidence indicates that the etiology, and thus risk factors, differ according to colorectal subsite and sex [4]. Specifically, proximal colon cancer has been suggested to be more common in females, whereas distal colon cancer is more common among males [5]. Furthermore, several modifiable risk factors have been identified. Smoking, body fatness, alcohol intake, and consumption of red and processed meat increase the risk, whereas physical activity and consumption of whole grains, dietary fiber, and calcium supplements decrease the risk of CRC [3].

In addition, other dietary components, such as sugar-sweetened beverages (SSBs), artificially sweetened beverages (ASBs), and juices, have been assessed for their role in CRC development. As these beverages are commonly consumed in large parts of the world, this knowledge is important for informing public health policies and generating new, or supporting existing, hypotheses about plausible mechanisms [6–8]. However, evidence from recent meta-analyses is inconsistent [9–15] and few prospective cohort studies have examined the associations between SSBs [16–25], ASBs [16, 17, 22, 23], and juice [16, 18, 23–25] and CRC risk. Of note, only five of these studies investigated cancer risk according to anatomical subsites of the colon [19, 20, 22, 24, 25], and only three used repeated measures of sweet beverage intake [21–23] in their analyses of CRC risk.

Although evidence is limited, plausible mechanisms underlying the associations between sweet beverages and CRC risk exist. SSBs are beverages containing added sugars and mainly consist of simple carbohydrates [26]. Consumption of SSBs increases the risk of body fatness and type 2 diabetes, both of which are risk factors for CRC [26–28], and may promote carcinogenesis through systemic inflammation, hyperinsulinemia, hyperglycemia, and alterations in the gut microbiota [29, 30]. Moreover, sugar may promote the growth and proliferation of cancer cells [31]. Additionally, some sodas contain the color additive 4-methylimidazole, which is proposed to have a carcinogenic effect [12, 16]. The consumption of SSBs has declined over the past two decades in Norway, with 44 L per capita sold in 2022. However, Norwegians still consumed more added sugar than the upper recommended limit of 10% of the total energy intake [32].

ASBs are beverages with added non-caloric sweeteners, such as aspartame, steviol glycosides, sucralose, and acesulfame K. The World Health Organization recently advised against using non-caloric sweeteners for weight management or to reduce the risk of non-communicable diseases, as emerging evidence suggests that long-term consumption can result in negative health outcomes, including an increased risk of obesity and type 2 diabetes [33]. In Norway, ASB sales have increased over the past two decades and surpassed those of SSBs in 2019 [32]. In 2022, 73 L of ASBs per person were sold.

In addition to color and flavor additives, ASBs are hypothesized to influence CRC development through inflammation and gut microbiota alterations [12]. Chronic inflammation and dysbiosis can promote carcinogenesis by oxidative stress, inflammatory signals, and increased epithelial permeability [34]. Pathogenic bacteria may also directly affect epithelial cells and suppress immune reactions in the tumor microenvironment [34]. Studies suggest that the sweeteners saccharin, sucralose, steviol glycosides, and polyols may affect the microbial composition, while aspartame and acesulfame K do not, but more human trials are needed [35]. Additionally, a proposed cell-damaging effect of artificial sweetener metabolites, with an emphasis on aspartame metabolites, has been hypothesized based on results from laboratory and animal studies [12]. However, the mechanistic evidence of a role for aspartame in carcinogenesis is limited [36].

Juices termed as 100% fruit juices do not contain added sugars or sweeteners. Although fruit juices contain vitamins, minerals, and varying amounts of fiber, they also provide simple carbohydrates and energy in amounts comparable to those in SSBs. Thus, high juice consumption may have many of the same consequences as high SSB consumption. In 2010–2011, the reported mean juice consumption in Norway was 114 and 100 g/day for males and females, respectively [37].

Given the limited evidence, the potential role of sweet beverages in CRC development, and the indications that risk factors may be subsite-specific, we used repeated measures to assess the associations between the intake of SSBs, ASBs, and juice and the risk of overall CRC and cancer at colorectal subsites among Norwegian women.

## Methods

### Study sample

The Norwegian Women and Cancer (NOWAC) Study is a population-based prospective study, representative of Norwegian women born between 1927 and 1965 [38, 39]. Participants were randomly sampled by Statistics Norway using their unique identity numbers listed in the National Population Register. From 1991 to 2007, 172,472 women responded to study invitations and self-administered questionnaires distributed via mail. The participants received up to three follow-up questionnaires. The response rates for the first questionnaires varied from 48 to 57%, whereas the response rate for the second questionnaire distributed from 1998 to 2002 was 82% [39]. The questionnaires were distributed in separate waves, and the lengths and questions differed. All questionnaires collected information on disease history, socioeconomic status, anthropometric measures, lifestyle factors, and reproductive health. However, a detailed food frequency questionnaire (FFQ) including questions on sweet beverage consumption, was first distributed in 1998.

In the present study, we defined the period from 1998 to 2004 as the baseline, during which the participants first reported their intake of SSBs, ASBs, and juice. The subsequent questionnaire collecting information on sweet beverage intake six-ten years later was used as the follow-up questionnaire. Consequently, 93,140 women aged 41–77 years at baseline were eligible for inclusion.

The NOWAC study was approved by the Regional Committee for Medical and Health Research Ethics (REK) and the Norwegian Data Protection Authority. Additionally, this study has an approved Data Protection Impact Assessment (no. 537703) and was approved by REK (no. 778138). Participants provided informed consent to participate and to link their data to the Cancer Registry of Norway and the Norwegian Cause of Death Registry. Data were processed on the basis of public interest.

### Exposures

Information on sweet beverage consumption was collected using validated self-administered semi-quantitative FFQs [40, 41]. Participants reported their usual intake of foods and beverages commonly consumed in a Norwegian diet in the preceding year. Nutrient consumption was calculated from the FFQ responses using official standardized portion sizes and weights [42] and the Norwegian food composition tables [43–46]. Participants responded to one question about SSB consumption (“how many glasses of soft drinks/squash with sugar do you usually drink?”) and one about ASB consumption (“how many glasses of sugar free soft drinks/squash do you usually drink?”). The FFQs differed slightly across waves regarding questions for juice consumption. The baseline questionnaires were limited to one question on orange juice consumption. In contrast, the follow-up questionnaires additionally asked about the intake of other juice types. For the follow-up questionnaires, we combined the intakes of orange juice and other juices into a single variable. Participants answered within a range from never/rarely to 4 + glasses per day. The intake of SSB, ASB and juice was divided into three groups: never/seldom, 1–6 glasses/week and ≥ 7 glasses/week. Additionally, we modeled the consumption of sweet beverages continuously by one glass increase per day. One glass of sweet beverage corresponded to 210 g.

### Exclusion criteria

**Fig. 1:**
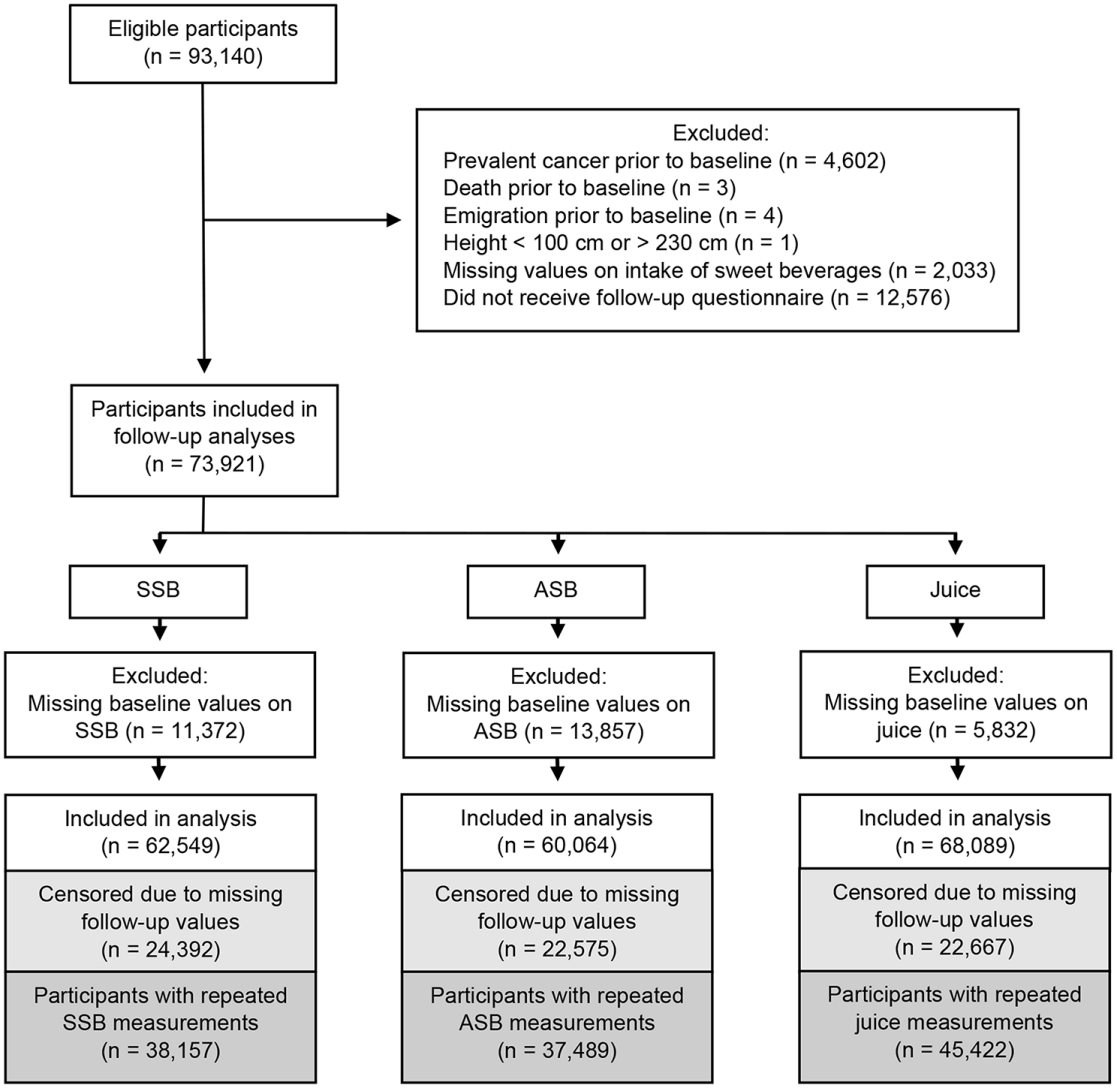
Flow chart of participants included in analyses of colorectal cancer risk

We excluded participants with prevalent cancer (except non-melanoma skin cancer) at baseline (*n* = 4,602), those who died (*n* = 3) or emigrated (*n* = 4) before the registration of baseline questionnaires, those with a height < 100 cm or > 230 cm (*n* = 1), or those with missing baseline values of SSB, ASB, and juice intake (*n* = 2,033) (Fig. 1). None of the participants had implausible weight values at baseline (< 30 kg or > 200 kg). Additionally, we excluded participants who did not receive a follow-up questionnaire (*n* = 12,576), all of whom were born prior to 1943. Therefore, 73,921 participants aged 41–61 years at baseline were included in the final study sample. Finally, for each analysis of SSB, ASB, and juice consumption, we excluded participants with missing information on baseline SSB (*n* = 11,372), ASB (*n* = 13,857), or juice intake (*n* = 5,832). Consequently, 62,549 participants were included in the SSB analysis, 60,064 in the ASB analysis and 68,089 in the juice analysis, respectively.

### Follow-up

Participants were followed from baseline until the date of emigration, death, diagnosis, censoring, or end of follow-up (December 18, 2018), whichever occurred first. Information on the dates of emigration and death was obtained through linkages with the National Population Register. Participants who did not return the follow-up questionnaire were censored on the date they could have responded, and those who did not report their intake of SSB, ASB, or juice were censored on the date the follow-up questionnaire was received (Fig. 1).

### Outcome ascertainment

The primary incident cases of CRC were ascertained through linkages to the Cancer Registry of Norway. CRC cases were identified using the International Classification of Diseases 10th Revision (ICD-10) codes C18-20 [47] and the International Classification of Diseases for Oncology 3rd edition (ICD-O-3) codes C19.9 and C20.9 [48]. We further categorized CRC into the following subgroups: colon cancer (ICD 10 C18-18.9), proximal colon cancer (ICD 10 C18.0-18.5), distal colon cancer (ICD 10 C18.6-18.7), and rectal cancer (ICD 10 C19-20; ICD-O-3 C19.9, and C20.9). Only CRC cases coded with ICD tumor behavior code 3 (ICD O3/3 malignant, primary site) and classified as adenocarcinomas (morphology codes 8140, 8210–8211, 8255, 8260–8263, 8480–8481, 8574, and 8470) were included [49] (Additional file 1: Table S1).

### Confounders

We constructed three directed acyclic graphs (DAGs) to identify confounders of the association between each sweet beverage type and CRC. First, we included nodes for, and paths between beverage consumption, colorectal cancer, and their common causes. We then added nodes for the common causes of existing nodes and their connecting paths. We identified the same confounders for all three DAGs: age, education, and diabetes status at baseline (Additional file 1: Fig. S1–S3).

Information on age was retrieved from the National Population Register and included as a continuous variable in the models. Information on prevalent diabetes (yes or no) was retrieved from our baseline questionnaires, whereas education level (< 10, 10–12, > 12 years) was collected from either baseline questionnaires or questionnaires completed before our baseline.

### Missing variables

We assumed that most participants with missing information on diabetes status at baseline did not have prevalent diabetes [50]. Thus, we recoded missing values on diabetes to “no diabetes”. If participants had missing values for education at baseline, they were excluded from the multivariable analyses.

### Statistical analyses

The study sample characteristics are presented as percentages of distributions, means with standard deviations, and medians with interquartile ranges. We used Cox proportional hazard regression with age as the underlying time metric to estimate the associations between sweet beverage consumption and overall CRC incidence and cancer at the colorectal subsites. Baseline values of sweet beverage consumption were used until follow-up data were available. Thereafter, the follow-up values were used. Effect estimates were presented as hazard ratios (HRs) with 95% confidence intervals (CIs). Linear trends were estimated by including SSB, ASB and juice as continuous variables in the regression models. We used the Wald test to assess heterogeneity between colon cancer and rectal cancer risk, and proximal colon cancer and distal colon cancer risk, both for analyses with a continuous exposure and between linear trends. The statistical significance level was set at *p* < 0.05. The proportional hazard assumption was assessed via visual inspection of Schoenfeld residuals and was satisfied. We presented two models: Model 1 adjusted for age and Model 2 additionally adjusted for education and diabetes status at baseline. The effect estimates should be interpreted as the total causal effect of the sweet beverage intake on CRC risk, as energy intake was considered a mediator and therefore not included in the model [51]. All analyses were performed using Stata 17.0 [52].

#### Sensitivity analyses

We performed five sensitivity analyses. First, we excluded participants with ≤ 2 years of follow-up due to the risk of reverse causation. Second, because participants with missing information on education at baseline were excluded from the multivariable-analyses, we performed age-adjusted analyses in the smaller Model 2 sample to evaluate whether changes in effect estimates from Model 1 to Model 2 were caused by the reduced sample size or the inclusion of education and diabetes status at baseline as covariates. Third, due to a large number of participants being censored during follow-up, we performed analyses where participants with missing follow-up information on sweet beverage intake had their baseline value of sweet beverage intake carried forward, and participants remained in the analyses until the date of CRC diagnosis, death, emigration, or end of follow-up. Fourth, we performed analyses by age groups (≤ 50y, > 50y), education levels (< 10, 10–12, > 12 years), and among participants not reporting a baseline diabetes diagnosis to investigate if the associations between sweet beverages and overall CRC risk differed between subgroups.

Lastly, to address the potential issue of residual confounding, we conducted analyses including probable and established risk factors for CRC collected from baseline questionnaires [3]. Smoking status was categorized as never, former, or current. Physical activity level was self-reported on a 10-point scale, with 1–3 classified as low, 4–7 as moderate, and 8–10 as high physical activity. Red meat consumption was divided into tertiles based on the summarized intake of beef, chops, and roasts. Similarly, processed meat was divided into tertiles based on the combined intake of cold cuts, sausages, and meatballs. Alcohol consumption was calculated from the intake of wine, beer, spirits, liqueur, and fortified wine, and categorized into three groups: 0 g/day, ≤ 6 g/day, and > 6 g/day. Intake of fiber and calcium was derived from the FFQ and modeled as continuous variables (g/day).

## Results

During a median follow-up of 16.5 years from baseline, 1,187 women were diagnosed with primary incident CRC, including 796 with colon cancer, 472 with proximal colon cancer, 308 with distal colon cancer, and 391 with rectal cancer. Of the participants with colon cancer, 1 participant was diagnosed with overlapping lesion of colon (ICD 10 C18.8) and 15 with unspecified colon cancer (ICD 10 C18.9), and these cases were not included in proximal colon cancer or distal colon cancer. The median age at diagnosis was 63 years for overall CRC, 64 years for colon cancer, 65 years for proximal colon cancer, and 62 years for distal colon cancer and rectal cancer. Table 1 presents the baseline characteristics of the study sample categorized according to the intake of SSB, ASB, and juice. Additional file 1: Table S2-S4 displays the proportion of participants who consumed SSBs, ASBs, and juice at baseline and follow-up.

**Table 1:** Baseline characteristics of the study sample according to SSB, ASB, and juice intake

	Intake of SSB			Intake of ASB			Intake of juice		
	Never/ seldom	1–6 glasses/ week	≥ 7 glasses/ week	Never/ seldom	1–6 glasses/week	≥ 7 glasses/week	Never/ seldom	1–6 glasses /week	≥ 7 glasses /week
**Number of participants**, ***n* (%)**	34,596 (55.3)	22,842 (36.5)	5,111 (8.2)	37,742 (62.8)	16,033 (26.7)	6,289 (10.5)	26,090 (38.3)	28,791 (42.3)	13,208 (19.4)
**Age (y)**,** mean (SD)**	51.1 (4.9)	49.7 (5.1)	49.4 (5.2)	50.8 (5.1)	50.0 (5.0)	50.0 (5.0)	50.9 (5.0)	50.4 (5.1)	50.7 (5.1)
**Height**^**1**^**(cm)**,** mean (SD)**	166.6 (5.7)	166.5 (5.6)	166.4 (5.7)	166.6 (5.7)	166.5 (5.6)	166.5 (5.6)	166.3 (5.7)	166.5 (5.6)	167.0 (5.6)
**Education (y)**,** %**									
< 10	28.6	33.4	39.1	28.4	34.4	35.8	35.5	29.7	26.0
10–12	22.6	23.4	23.6	22.5	23.5	24.8	23.6	23.1	21.9
> 12	48.8	43.2	37.3	49.1	42.1	39.4	40.9	47.2	52.1
**Diabetes**,** %**	2.3	0.3	0.2	0.8	3.4	5.7	2.6	1.0	0.7
**Alcohol**^**1**^**(g/day)**,** %**									
0	14.4	20.4	25.2	15.8	17.0	20.4	20.2	15.2	13.1
≤ 6	60.8	61.6	58.1	60.6	63.2	59.5	60.3	62.8	60.3
> 6	24.8	18.0	16.7	23.6	19.8	20.1	19.5	22.0	26.6
**Smoking status**^**1**^, **%**									
Never	35.1	40.6	35.1	37.7	35.6	31.3	33.5	39.4	38.7
Former	37.3	29.9	25.4	34.4	36.0	33.3	34.3	33.5	35.3
Current	27.6	29.5	39.5	27.9	28.4	35.4	32.2	27.1	26.0
**BMI**^**1**^**(kg/m²)**,** %**									
Underweight (< 18.5)	1.2	1.3	2.5	1.4	0.7	1.0	1.4	1.2	1.4
Normal weight (18.5–24.9)	58.2	61.8	62.9	64.3	48.1	42.7	55.5	60.2	65.3
Overweight (25.0-29.9)	30.6	28.4	26.6	27.3	36.5	35.7	31.3	30.1	26.6
Obesity (≥ 30)	10.0	8.5	8.0	7.0	14.7	20.6	11.8	8.5	6.7
**Physical activity**^**1**^, **%**									
Low	11.5	11.5	14.7	10.8	12.2	17.8	13.7	10.3	9.9
Moderate	72.4	75.4	71.7	73.6	74.0	69.6	71.6	75.0	73.6
High	16.1	13.1	13.6	15.6	13.8	12.6	14.7	14.7	16.5
**Calcium**^**1**^**(mg/day)**,** median (iqr)**	664 (394)	719 (425)	722 (451)	683 (409)	684 (408)	678 (406)	648 (420)	700 (402)	716 (401)
**Fiber**^**1**^**(g/day)**,** median (iqr)**	21 (9)	21 (8)	21 (9)	21 (9)	21 (9)	21 (9)	20 (9)	22 (8)	22 (9)
**Red meat**^**1**^**(g/day)**,** %**									
Low (0)	36.5	30.5	28.2	36.1	30.8	28.6	36.1	31.8	34.0
Medium (> 0–≤ 17.7)	33.7	35.9	33.1	34.2	34.9	32.5	33.3	35.1	34.0
High (> 17.7)	29.8	33.6	38.7	29.7	34.3	38.9	30.6	33.1	32.0
**Processed meat**^**1**^**(g/day)**,** %**									
Low (≤ 4.1)	39.2	25.2	22.2	36.3	28.7	26.4	34.5	32.0	35.1
Medium (> 4.1–≤ 39.2)	32.6	36.0	31.3	32.8	35.5	31.8	32.1	34.8	33.2
High (> 39.2)	28.2	38.8	46.5	30.9	35.8	41.8	33.4	33.2	31.7
**SSB**									
Never/seldom	N/A	N/A	N/A	63.9	55.9	69.9	65.5	51.5	53.7
1–6 glasses/week	N/A	N/A	N/A	30.3	41.0	18.2	28.6	42.1	35.3
≥ 7 glasses/week	N/A	N/A	N/A	5.8	3.1	11.9	5.9	6.4	11.0
**ASB**									
Never/seldom	70.7	65.6	71.7	N/A	N/A	N/A	68.6	63.1	65.3
1–6 glasses/week	21.1	30.2	12.9	N/A	N/A	N/A	22.7	29.4	23.1
≥ 7 glasses/week	8.2	4.2	15.4	N/A	N/A	N/A	8.7	7.5	11.6
**Juice**									
Never/seldom	47.3	33.8	35.3	43.8	37.5	42.1	N/A	N/A	N/A
1–6 glasses/week	36.7	49.0	37.7	39.0	46.9	35.0	N/A	N/A	N/A
≥ 7 glasses/week	16.0	17.2	27.0	17.2	15.6	22.9	N/A	N/A	N/A
**Energy**^**1**^**(kJ)**,** median (iqr)**	6702 (2339)	7377 (2460)	7977 (2658)	7027 (2463)	6825 (2400)	6800 (2522)	6573 (2382)	7127 (2413)	7555 (2483)

^1^Height was self-reported; alcohol consumption was calculated from the intake of wine, beer, spirits, liqueur, and fortified wine reported in FFQs and an intake < or > 6 g was used as cut-off points; smoking status was self-reported; BMI was calculated using self-reported height and weight; physical activity was self-reported and based on a scale from 1–10, where 1–3 was classified as low, 4–7 as moderate, and 8–10 as high physical activity; intake of calcium and fiber was calculated from FFQs; red meat consumption is tertiles of the summarized intake of beef, chops, and roasts and processed meat is tertiles based on the sum of cold cuts, sausages, and meatballs reported in the FFQs; energy intake was calculated from the FFQs

Abbreviations: y = years, SD = standard deviation, g/day = grams per day, BMI = body mass index, mg/day = milligrams per day, iqr = interquartile range (25th− 75th percentile), kJ = kilojoules. 1 glass = 210 ml

Compared to non-consumers and low consumers of SSB, high consumers of SSB were slightly younger, less educated, less physically active, more likely to be current smokers, had a lower prevalence of diabetes, overweight, and obesity, consumed less alcohol, and had a higher intake of energy and red and processed meat. Further, high consumers of ASB reported fewer years of education, lower levels of physical activity, higher prevalence of diabetes and obesity, higher consumption of red and processed meat, and were more likely to abstain from alcohol and be current smokers than women with a lower ASB intake. Participants who consumed ≥ 7 glasses of juice per week were slightly taller, more educated, more physically active, less likely to be current smokers, had a lower prevalence of diabetes, overweight, and obesity, and had a higher intake of alcohol, calcium, and energy compared to participants who consumed less juice. Finally, a larger proportion of high consumers of one type of sweet beverage were also high consumers of other sweet beverages compared to participants with lower sweet beverage intake.

### SSB and risk of colorectal cancer

After adjusting for age, education, and diabetes status at baseline, we observed no associations between SSB consumption and risk of total CRC (HR_1 − 6 glasses/wk_ = 0.87, 95% CI: 0.73–1.04; HR_≥ 7 glasses/wk_ = 1.15, 95% CI: 0.84–1.59; *p*-trend = 0.698), colon cancer (HR_1 − 6 glasses/wk_ = 0.95, 95% CI: 0.77–1.19; HR_≥ 7 glasses/wk_ = 1.07, 95% CI: 0.70–1.62; *p*-trend = 0.939), proximal colon cancer (HR_1 − 6 glasses/wk_ = 1.08, 95% CI: 0.81–1.43; HR_≥ 7 glasses/wk_ = 0.99, 95% CI: 0.55–1.79; *p*-trend = 0.748), or distal colon cancer (HR_1 − 6 glasses/wk_ = 0.86, 95% CI: 0.60–1.22; HR_≥ 7 glasses/wk_ = 1.11, 95% CI: 0.59–2.06; *p*-trend = 0.746) when comparing women who consumed 1–6 glasses/week or ≥ 7 glasses/week to women who never or seldom drank SSBs (Table 2). In contrast, compared to no consumption, consuming 1–6 glasses/week of SSB indicated a reduced risk of rectal cancer (HR = 0.73, 95% CI: 0.53-1.00). However, consuming ≥ 7 glasses/week was not associated with rectal cancer risk (HR = 1.30, 95% CI: 0.79–2.16) and no linear trend was observed (*p*-trend = 0.585). Additionally, we observed no heterogeneity between subsites, and an increase of 210 ml SSB per day showed no associations with the risk of total or subsite-specific CRC (Additional file 1: Table S5).

**Table 2:** Associations between SSB intake and risk of overall and subsite-specific colorectal cancer

	Model 1					Model 2					
	*n*/cases	HR	95% CI	*p*-value	*p*-trend	*n*/cases	HR	95% CI	*p*-value	*p*-trend	*p*-het
**Total CRC**	62,549/714					60,259/687					
Never/seldom	42,464/505	1.00				40,865/486	1.00				
1–6 glasses/week	16,595/166	0.88	0.74–1.05	0.154		16,012/160	0.87	0.73–1.04	0.136		
≥ 7 glasses/week	3,490/43	1.19	0.87–1.63	0.275		3,382/41	1.15	0.84–1.59	0.386		
					0.838					0.698	
**Colon cancer**	62,549/464					60,259/448					
Never/seldom	42,464/324	1.00				40,865/313	1.00				
1–6 glasses/week	16,595/116	0.98	0.79–1.22	0.860		16,012/111	0.95	0.77–1.19	0.677		
≥ 7 glasses/week	3,490/24	1.06	0.70–1.61	0.775		3,382/24	1.07	0.70–1.62	0.768		
					0.942					0.939	
**Proximal colon cancer**	62,549/272					60,259/262					
Never/seldom	42,464/189	1.00				40,865/182	1.00				
1–6 glasses/week	16,595/71	1.10	0.83–1.45	0.507		16,012/68	1.08	0.81–1.43	0.615		
≥ 7 glasses/week	3,490/12	0.98	0.54–1.76	0.940		3,382/12	0.99	0.55–1.79	0.983		
					0.691					0.748	
**Distal colon cancer**	62,549/182					60,259/176					
Never/seldom	42,464/126	1.00				40,865/122	1.00				
1–6 glasses/week	16,595/45	0.89	0.63–1.26	0.527		16,012/43	0.86	0.60–1.22	0.396		
≥ 7 glasses/week	3,490/11	1.14	0.61–2.11	0.687		3,382/11	1.11	0.59–2.06	0.746		
					0.887					0.746	0.131^1^
**Rectal cancer**	62,549/250					60,259/239					
Never/seldom	42,464/181	1.00				40,865/173	1.00				
1–6 glasses/week	16,595/50	0.71	0.52–0.97	0.031		16,012/49	0.73	0.53-1.00	0.052		
≥ 7 glasses/week	3,490/19	1.40	0.87–2.25	0.165		3,382/17	1.30	0.79–2.16	0.301		
					0.661					0.585	0.777^2^

^1^*p*-value for heterogeneity between proximal and distal colon cancer

^2^*p*-value for heterogeneity between colon cancer and rectal cancer

Model 1: Adjusted for age

Model 2: Adjusted for age, education, and diabetes status at baseline

Abbreviations: *n* = number of observations, HR = hazard ratio, CI = confidence interval, CRC = colorectal cancer

1 glass corresponds to 210 ml

### ASB and risk of colorectal cancer

Compared to women who never or seldom consumed ASB, we observed a positive association between ASB and colon cancer risk among participants who consumed 1–6 glasses/week (HR = 1.26, 95% CI: 1.02–1.55) after adjusting for age, education, and diabetes status at baseline (Table 3). In contrast, this was not observed for participants who consumed ≥ 7 glasses/week (HR = 1.01, 95% CI: 0.72–1.42) and the *p*-trend was non-significant (*p*-trend = 0.246). Moreover, the intake of ASB was not associated with proximal colon cancer (HR_1 − 6 glasses/wk_ = 1.25, 95% CI: 0.94–1.65; HR_≥ 7 glasses/wk_ = 1.11, 95% CI: 0.72–1.71; *p*-trend = 0.246), distal colon cancer (HR_1 − 6 glasses/wk_ = 1.31, 95% CI: 0.95–1.81; HR_≥ 7 glasses/wk_ = 0.95, 95% CI: 0.55–1.63; *p*-trend = 0.494), rectal cancer (HR_1 − 6 glasses/wk_ = 0.91, 95% CI: 0.67–1.25; HR_≥ 7 glasses/wk_ = 0.76, 95% CI: 0.46–1.28; *p*-trend = 0.278), or total CRC (HR_1 − 6 glasses/wk_ = 1.14, 95% CI: 0.96–1.35; HR_≥ 7 glasses/wk_ = 0.92, 95% CI: 0.70–1.23; *p*-trend = 0.746). Similarly, no associations with total CRC or subsite-specific CRC were observed after modeling ASB as a continuous variable (Additional file 1: Table S5). Further, the linear trend of colon cancer risk was significantly different from that of rectal cancer risk (*p*-heterogeneity = 0.037). However, no difference was observed when ASB was modeled as a continuous variable (*p*-heterogeneity = 0.356).

**Table 3:** Associations between ASB intake and risk of overall and subsite-specific colorectal cancer

	Model 1					Model 2					
	*n*/cases	HR	95% CI	*p*-value	*p*-trend	*n*/cases	HR	95% CI	*p*-value	*p*-trend	*p*-het
**Total CRC**	60,064/722					57,858/692					
Never/seldom	40,064/474	1.00				38,596/457	1.00				
1–6 glasses/week	14,367/192	1.17	0.99–1.38	0.067		13,837/180	1.14	0.96–1.35	0.148		
≥ 7 glasses/week	5,633/56	0.91	0.69–1.20	0.485		5,425/55	0.92	0.70–1.23	0.587		
					0.683					0.746	
**Colon cancer**	60,064/479					57,858/463					
Never/seldom	40,064/304	1.00				38,596/295	1.00				
1–6 glasses/week	14,367/135	1.29	1.06–1.59	0.013		13,837/129	1.26	1.02–1.55	0.030		
≥ 7 glasses/week	5,633/40	1.02	0.73–1.42	0.905		5,425/39	1.01	0.72–1.42	0.939		
					0.173					0.246	
**Proximal colon cancer**	60,064/278					57,858/268					
Never/seldom	40,064/177	1.00				38,596/172	1.00				
1–6 glasses/week	14,367/76	1.28	0.98–1.68	0.069		13,837/72	1.25	0.94–1.65	0.119		
≥ 7 glasses/week	5,633/25	1.13	0.74–1.72	0.560		5,425/24	1.11	0.72–1.71	0.627		
					0.169					0.246	
**Distal colon cancer**	60,064/192					57,858/186					
Never/seldom	40,064/120	1.00				38,596/116	1.00				
1–6 glasses/week	14,367/57	1.34	0.97–1.83	0.073		13,837/55	1.31	0.95–1.81	0.111		
≥ 7 glasses/week	5,633/15	0.93	0.54–1.59	0.782		5,425/15	0.95	0.55–1.63	0.846		
					0.479					0.494	0.105^1^
**Rectal cancer**	60,064/243					57,858/229					
Never/seldom	40,064/170	1.00				38,596/162	1.00				
1–6 glasses/week	14,367/57	0.95	0.71–1.29	0.751		13,837/51	0.91	0.67–1.25	0.573		
≥ 7 glasses/week	5,633/16	0.71	0.42–1.18	0.183		5,425/16	0.76	0.46–1.28	0.309		
					0.229					0.278	0.037^2^

^1^*p*-value for heterogeneity between proximal and distal colon cancer

^2^*p*-value for heterogeneity between colon cancer and rectal cancer

Model 1: Adjusted for age

Model 2: Adjusted for age, education, and diabetes status at baseline

Abbreviations: *n* = number of observations, HR = hazard ratio, CI = confidence interval, CRC = colorectal cancer

1 glass corresponds to 210 ml

### Juice and risk of colorectal cancer

Compared to participants who never or seldom consumed juice, we observed a reduced risk of total CRC (HR_1 − 6 glasses/wk_ = 0.88, 95% CI: 0.76–1.03; HR_≥ 7 glasses/wk_ = 0.81, 95% CI: 0.67–0.98; *p*-trend = 0.025), colon cancer (HR_1 − 6 glasses/wk_ = 0.91, 95% CI: 0.75–1.10; HR_≥ 7 glasses/wk_ = 0.73, 95% CI: 0.58–0.94; *p*-trend = 0.015), and proximal colon cancer (HR_1 − 6 glasses/wk_ = 0.98, 95% CI: 0.76–1.25; HR_≥ 7 glasses/wk_ = 0.71, 95% CI: 0.52–0.99; *p*-trend = 0.065) among juice consumers after adjusting for age, education, and diabetes status at baseline (Table 4). There was no clear association between juice consumption and risk of distal colon cancer (HR_1 − 6 glasses/wk_ = 0.83, 95% CI: 0.61–1.11; HR_≥ 7 glasses/wk_ = 0.76, 95% CI: 0.53–1.11; *p*-trend = 0.118), or rectal cancer (HR_1 − 6 glasses/wk_ = 0.83, 95% CI: 0.63–1.10; HR_≥ 7 glasses/wk_ = 0.97, 95% CI: 0.71–1.34; *p*-trend = 0.676). Further, no significant heterogeneity was observed between colon cancer and rectal cancer, or proximal colon cancer and distal colon cancer.

**Table 4:** Associations between fruit juice intake and risk of overall and subsite-specific colorectal cancer

	Model 1					Model 2					
	*n*/cases	HR	95% CI	*p*-value	*p*-trend	*n*/cases	HR	95% CI	*p*-value	*p*-trend	*p*-het
**Total CRC**	68,089/822					65,391/791					
Never/seldom	27,709/363	1.00				26,597/345	1.00				
1–6 glasses/week	26,075/304	0.86	0.74-1.00	0.055		25,031/295	0.88	0.76–1.03	0.123		
≥ 7 glasses/week	14,305/155	0.79	0.65–0.95	0.013		13,763/151	0.81	0.67–0.98	0.034		
					0.008					0.025	
**Colon cancer**	68,089/547					65,391/529					
Never/seldom	27,709/243	1.00				26,597/233	1.00				
1–6 glasses/week	26,075/211	0.90	0.75–1.08	0.252		25,031/204	0.91	0.75–1.10	0.328		
≥ 7 glasses/week	14,305/93	0.71	0.56–0.90	0.004		13,763/92	0.73	0.58–0.94	0.013		
					0.005					0.015	
**Proximal colon cancer**	68,089/316					65,391/304					
Never/seldom	27,709/138	1.00				26,597/131	1.00				
1–6 glasses/week	26,075/127	0.97	0.76–1.23	0.785		25,031/122	0.98	0.76–1.25	0.849		
≥ 7 glasses/week	14,305/51	0.68	0.49–0.94	0.020		13,763/51	0.71	0.52–0.99	0.042		
					0.034					0.065	
**Distal colon cancer**	68,089/220					65,391/214					
Never/seldom	27,709/100	1.00				26,597/97	1.00				
1–6 glasses/week	26,075/80	0.81	0.60–1.09	0.160		25,031/78	0.83	0.61–1.11	0.212		
≥ 7 glasses/week	14,305/40	0.74	0.51–1.06	0.104		13,763/39	0.76	0.53–1.11	0.157		
					0.073					0.118	0.665^1^
**Rectal cancer**	68,089/275					65,391/262					
Never/seldom	27,709/120	1.00				26,597/112	1.00				
1–6 glasses/week	26,075/93	0.79	0.60–1.03	0.085		25,031/91	0.83	0.63–1.10	0.196		
≥ 7 glasses/week	14,305/62	0.95	0.70–1.29	0.754		13,763/59	0.97	0.71–1.34	0.869		
					0.516					0.676	0.526^2^

^1^*p*-value for heterogeneity between proximal and distal colon cancer

^2^*p*-value for heterogeneity between colon cancer and rectal cancer

Model 1: Adjusted for age

Model 2: Adjusted for age, education, and diabetes status at baseline

Abbreviations: *n* = number of observations, HR = hazard ratio, CI = confidence interval, CRC = colorectal cancer

1 glass corresponds to 210 ml

Additionally, for each 210 ml daily increase in juice intake, we found a suggested inverse association with the risk of total CRC (HR = 0.90, 95% CI: 0.79–1.03) and cancer of the colon (HR = 0.85, 95% CI: 0.72-1.00), including both proximal colon cancer (HR = 0.88, 95% CI: 0.71–1.09) and distal colon cancer (HR = 0.81, 95% CI: 0.62–1.06), but not for rectal cancer (Additional file 1: Table S5). However, there were no indications of heterogeneity between subsites.

### Sensitivity analyses

After excluding participants with ≤ 2 years of follow-up, we observed results similar to those of the main analyses (Additional file 1: Table S6-S8). Additionally, the age-adjusted analyses in the smaller Model 2 sample did not alter our observations, indicating that changes in the HRs from Model 1 to Model 2 are mainly explained by the included covariates (Additional file 1: Table S9). Similarly, our interpretation of the results did not change after carrying forward the baseline value of sweet beverage intake for participants with missing follow-up data (Additional file 1: Table S10-S12),

Further, after stratifying by baseline age, we observed no difference between the groups in the analyses of SSBs and ASBs. Contrarily, a reduced risk of total CRC was observed among juice consumers ≤ 50 years of age (*p*-trend = 0.046), but not among older participants (Additional file 1: Table S13).

Examining the association between SSBs, ASBs and CRC risk by education level showed no linear trend in any of the strata (Additional file 1: Table S13). However, an inverse association was observed between juice intake and CRC risk among participants with > 12 years of education (*p*-trend = 0.043), but not for participants with fewer years of education. Additionally, results from the analyses excluding participants with diabetes at baseline were similar to our main analyses (Additional file 1: Table S13).

Finally, in the sensitivity analyses additionally adjusting for probable and established risk factors for CRC (Additional file 1: Table S14), there were small changes to the effect estimates, but the overall interpretation remained unchanged. Specifically, the suggested decreased risk of rectal cancer among consumers of 1–6 glasses/week of SSB was no longer significant (HR = 0.76, 95% CI: 0.53–1.07). The same was observed for the suggested increased risk of colon cancer among consumers of 1–6 glasses of ASB per week (HR = 1.21, 95% CI: 0.97–1.50). Lastly, the associated reduced risk of proximal colon cancer among daily consumers of juice became non-significant after adjusting for potential confounders (HR = 0.75, 95% CI: 0.54–1.06).

## Discussion

We assessed associations between the consumption of SSBs, ASBs, or 100% fruit juice and the risk of overall and subsite-specific CRC. We observed no substantial associations between the intake of SSBs or ASBs and CRC risk. However, juice consumption was inversely associated with CRC risk. Specifically, women who consumed ≥ 7 glasses of juice per week had a respective 19% and 27% decreased risk of overall CRC and colon cancer compared to non-consumers. Moreover, the analyses of cancers of the proximal and distal colon showed a non-significant inverse trend. However, no evident association was observed between juice intake and cancer of the rectum.

Previously, decreased odds of CRC and colon cancer associated with juice intake have been observed in case-control studies in Egypt and Saudi Arabia, respectively [53, 54]. However, other observational studies found no evident association between juice intake and the odds of overall CRC [24, 55] or colon cancer [25, 56]. In contrast, some studies reported increased odds of overall CRC [56–58] and rectal cancer [56]. Furthermore, one study suggested increased odds of colon cancer among fruit juice consumers of both sexes [59], and another study suggested increased odds of rectal cancer among females who consumed fruit juice, but not males [60]. Lastly, a recent meta-analysis did not find an association between juice intake and CRC risk when comparing high consumers with low consumers of juice (RR = 1.16, 95% CI: 0.92–1.46) or per 250 ml daily increase in intake (RR = 1.21, 95% CI: 1.00-1.47); however, the certainty of evidence was evaluated as very low [9]. Thus, current evidence is generally limited and inconclusive.

The mixed results from previous studies may be explained by the varying amounts of juice consumed across cohorts, as the sugar content of fruit juices is comparable to that of SSBs. However, a juice intake of ≥ 7 glasses/week (≥ 210 ml/day) among high consumers in our study is somewhat comparable to the high consumers in three of the studies that found positive associations between juice consumption and odds for CRC [56, 58, 59]. Hence, the sugar content of fruit juice alone cannot explain the contrasting results of our study. However, the use of different definitions of fruit juice is a more likely explanation for the observed differences. In our baseline questionnaires, we specifically asked participants about their orange juice intake, and questions on the intake of other juices were included only in the FFQs at follow-up. In contrast, other studies have included fruit drinks that may contain added sugar in their definition of fruit juice.

Although fruit juice contains sugars, it is also a dietary source of micronutrients and varying amounts of fiber and may thus have a protective effect against CRC development, both systemically and in local tissues, and different nutrients may act synergistically [61]. Specifically, carotenoids, potassium, vitamin C, and vitamin E protect against reactive oxygen species [3, 61, 62], while anthocyanins and flavonoids may decrease levels of C-reactive protein and E-selectin [61]. Additionally, there is suggestive evidence that foods containing vitamin C decrease the risk of colon cancer [3], while B vitamins, particularly folate, play an important role in genomic maintenance [62]. Several mechanisms have been proposed for how dietary fiber might prevent CRC [3, 4]. However, the contribution of fiber through fruit juice in our sample is likely insignificant, since only 3.5% of the participants in our study consumed > 7 glasses of 210 ml juice a week at baseline.

In our subsite-specific analyses, we observed a significant inverse association between juice intake and risk of colon cancer, but not rectal cancer. Although we cannot conclude if the cancer risk differs between these subsites due to few rectal cancer cases and no indication of heterogeneity, the nutrients in fruit juice may affect the segments differently. The colon could be exposed to beneficial nutrients not absorbed in the small intestine. Additionally, pH and the presence of different bacteria and short-chain fatty acids varies across the large intestine and rectum [5].

Moreover, results from our main analyses suggested that daily juice consumers had a reduced risk of proximal colon cancer. Tendencies were similar, though not significant, for distal colon cancer. This may be a result of insufficient statistical power as only 39 cases of distal colon cancer consumed ≥ 7 glasses of juice a week. An alternative explanation could be that proximal colon cancer often develops through the microsatellite instable (MSI)- or the CpG island methylator phenotype (CIMP) pathway, while distal colon cancer often follows the chromosomal instability (CIN) pathway [5, 63]. Nutrients in fruit juice may protect against mutations in DNA repair enzymes (as seen in the MSI/CIMP pathways) but not mutations in genes involved in cell growth (as seen in the CIN pathway). Interestingly, Song et al. [64] observed an inverse association between omega-3 polyunsaturated fatty acids and MSI-high tumors, and suggested a protective role in CRC carcinogenesis via DNA repair enzymes. However, due to overlapping definitions, tumors may display traits from several pathways [34]. Although our observation of a decreased risk of proximal colon cancer among daily juice consumers is novel, we did not find strong indications of subsite-specific risk-differences. However, the potential for such associations warrants further investigation.

We observed no evident association between SSB intake and the risk of CRC. In contrast, a meta-analysis of eight observational studies observed a 10% (95% CI: 1.04–1.15) increased risk of CRC per 250 ml/day increase of SSB intake and when comparing high consumers to low consumers [9]. Similarly, five additional studies also observed a positive association between SSB intake and the risk of overall CRC [24, 53, 57, 65, 66]. Furthermore, three studies, including a Mendelian randomization study, found a positive association between SSB consumption and colon cancer [20, 24, 67] and one study found a positive association between SSB intake and rectal cancer [24]. In contrast, five studies found no association between SSB consumption and overall CRC [20, 24, 68], colon cancer [24, 68], or rectal cancer [20, 24, 60, 67]. Thus, the evidence is inconsistent but indicates that a positive association between SSB consumption and CRC risk may exist.

Similar to our observations, four observational studies found no evident association between ASB intake and the risk of overall CRC [16, 17, 23] or rectal cancer [60]. However, when three of these studies [16, 17, 23] were included in a meta-analysis, a 22% (0.62–0.99) reduced risk of CRC with moderate risk of bias and low certainty was observed when comparing high consumers to low consumers of ASB [15]. Interestingly, studies that assessed the substitution of one serving of SSB with one serving of ASB observed a 17% decreased risk of early onset CRC in women from the Nurses’ Health Study (NHS) II [23] and an 18.7% reduction in proximal colon cancer incidence in both sexes from the NHS and the Health Professionals Follow-Up Study [22]. This indicates that ASBs do not reduce the risk of CRC per se, but that replacing SSB might reduce the risk. The authors of these studies [22, 23] suggested that unabsorbed fructose may promote colorectal carcinogenesis by increasing gut permeability and promoting tumor growth in the colon. Furthermore, except for the substitution study [22], we found no other studies assessing the association between ASB intake and the risk of cancer in colon subsites. In summary, existing research on ASB consumption and the risk of CRC or cancer in colorectal subsites is scarce and the results are inconclusive.

Explanations for the opposing results between studies assessing SSB and ASB consumption could be the different definitions of SSBs [69] and ASBs, the use of high-fructose corn syrup instead of sucrose in SSBs in some countries, different cut-off points and number of participants in comparison groups. Further, the risk could differ between males and females, across subsites, and depend on whether the CRC is defined as early- or late-onset CRC.

### Strengths

The strengths of this study include its prospective design, large cohort size, and a median follow-up period of 16.5 years. This allowed for the estimation of both the risk of overall CRC and cancer risk at the anatomical subsites in the colon and rectum. Furthermore, we had repeated measures of the exposure to better capture the long-term intake of sweet beverages, as the intake may change over time. This is of special importance, considering that more ASBs have become available since the collection of our baseline measures in 1998–2004 [32]. Finally, incident cancer cases were ascertained from the Cancer Registry of Norway, where the completeness of colon cancer and rectal cancer was respectively reported as 99.7% and 99.9%, and 94.3% of colon cancer cases and 97.3% of rectal cancer cases were morphologically verified for the period of 2018–2022 [70].

### Limitations

Our study has several limitations. First, generalization of our observations to the general female population in Norway should be done with caution. Our study sample consisted of middle-aged women predominantly born in Norway, and the external validity of the NOWAC Study was examined and deemed satisfactory in 2003 [38]. However, the Norwegian population has changed since then. For example, the proportion of first-generation immigrants increased from 6 to 16% between 2003 and 2023 [71, 72], and 9% of all cancer cases in Norway between 2018 and 2022 were diagnosed among immigrants [70]. Additionally, we censored 35% of our participants due to missing follow-up data on sweet beverage intake. However, the analyses with baseline values carried forward indicated that these participants were not notably different from the rest of the study sample.

Second, because of the design of our FFQs and the few high consumers of SSB, ASB, or juice in our sample, we could only assess the CRC risk associated with sweet beverage consumption at 0-1470 ml/week. However, except for a slightly lower juice intake, the NOWAC participants had a similar intake of sweet beverages as Norwegian women. In our sample, 9, 12, and 24% reported daily consumption of SSB, ASB, and juice, respectively, whereas 8, 13, and 32% of Norwegian women reported drinking SSB, ASB, or juice daily in 2012 [73].

Third, the consumption of SSBs and juice has decreased since 2012, while the intake of ASBs has increased, suggesting a shift in the overall consumption patterns in Norway [32, 74]. Our follow-up data, collected from 2004 to 2014, may not reflect these changes. However, a national dietary survey conducted from 2022 to 2023 showed that ASB consumption among women decreases with age, and that men > 40 years of age consume more ASBs than women in the same age group [74]. Thus, the increased ASB consumption in Norway could be attributed to changes in consumption patterns among groups other than women > 40 years of age. Additionally, the sweet beverage products available have changed since the time of the data collection, including beverages containing both artificial sweeteners and sucrose, which our FFQs may not have captured. However, our results are relevant to female Norwegians with similar characteristics and intake patterns. Moreover, as the development of invasive CRC from polyps usually takes a minimum of 10 years [5], it is uncertain how this change in beverage intake since 2012 may have affected the development of CRC cases diagnosed up until December 2018.

Fourth, the use of self-reported data may have caused measurement errors and, thus, misclassification of sweet beverage intake. For example, the participants might not have distinguished between 100% fruit juices and nectars containing added sugar. Although the reproducibility of the FFQ used in the NOWAC Study is relatively high and is comparable to similar cohort studies, with a Spearman correlation coefficient (*r*) of 0.70 for soft drinks and orange juice [41], the ranking ability was moderate for orange juice (*r =* 0.56) and soft drink (*r =* 0.49) consumption when compared to repeated 24 h dietary recalls [40].

Fifth, the use of DAGs to identify adjustment variables, in addition to measurement errors in these variables, may have resulted in residual confounding [75]. We based our DAGs on existing literature, and we had to make some assumptions about causal associations. Adjusting for age, education, and diabetes status at baseline may not have been sufficient to account for all confounding pathways between sweet beverage intake and CRC risk. The juice consumers in our sample had an overall healthier lifestyle at baseline compared to participants who consumed less juice. Moreover, at the time of data collection, one glass of 100% fruit- or vegetable juice daily was recommended by the Norwegian health authorities [76]. Thus, it is probable that our observations reflect an overall healthy lifestyle and not juice consumption itself. However, our analysis additionally adjusting for established risk factors for CRC, did not change the interpretation of our result, indicating that a healthy lifestyle does not completely explain the associations found.

Further, energy intake is often included as a covariate to reduce measurement error in epidemiological studies [77]. However, we chose not to include energy intake in our DAGs, as we considered it to be a mediator between the sweet beverages and CRC. Additionally, adjusting for total energy would alter the interpretation of our results by creating a substitution effect [51]. Still, measurement errors and residual confounding could have affected the associations in any direction [75].

Additionally, our analyses stratified by age at baseline, education level and diabetes status overall support our observations. However, these analyses indicated that the association between juice consumption and CRC risk may vary with age at baseline and education level. Nevertheless, due to the small number of observations, we do not want to draw any conclusion about potential differences between the groups.

Sixth, although our questionnaires collected data on important covariates, we did not have information on the type of diabetes, family history of CRC, history of inflammatory bowel disease, use of nonsteroidal anti-inflammatory drugs, or intake of energy drinks, which could potentially confound the association between sweet beverage intake and risk of CRC. Type 2 diabetes incidence is most likely a mediator between sweet beverage consumption and CRC risk, whereas prevalent type 1 and type 2 diabetes are more likely to be common causes of both sweet beverage consumption and CRC and are thus confounding factors. Incident type 1 diabetes is most likely an ancestor of the outcome. However, by including both the prevalence and incidence of diabetes in our DAGs, these differences could be partly accounted for. Additionally, since only 3.4 L per person of energy drinks were sold in Norway in 2014 [32], the potential confounding from energy drinks is likely to be minimal.

Finally, as this was an exploratory study, we did not adjust for multiple comparisons. However, as we conducted > 30 analyses, false positive findings may exist, and the results should be interpreted with this in mind.

In conclusion, we observed no substantial association between the intake of SSBs or ASBs and the risk of CRC or cancer in colorectal subsites in our cohort of Norwegian women. Conversely, our results indicate that juice consumption is associated with a reduced risk of CRC, particularly in the colon. These results warrant further investigation in larger cohorts with power to detect possible differences in cancer risk across colorectal subsites, especially as patterns of sweet beverage consumption are changing.

## Electronic supplementary material

Below is the link to the electronic supplementary material.

Supplementary Material 1: Figure S1: Directed acyclic graph for the association between SSB consumption and colorectal cancer incidence. Figure S2: Directed acyclic graph for the association between ASB consumption and colorectal cancer incidence. Figure S3: Directed acyclic graph for the association between juice consumption and colorectal cancer incidence. Table S1: Morphology codes (ICD-O-3) of adenocarcinomas for the cases in this study. Table S2: Cross-tabulation of SSB intake at baseline and follow-up. Table S3: Cross-tabulation of ASB intake at baseline and follow-up. Table S4: Cross-tabulation of juice intake at baseline and follow-up. Table S5: Associations between sweet beverage intake and risk of overall and subsite-specific colorectal cancer per 1 glass (250 ml) daily increase. Table S6: Associations between SSB intake and risk of overall and subsite-specific colorectal cancer for participants with > 2 years of follow up. Table S7: Associations between ASB intake and risk of overall and subsite-specific colorectal cancer for participants with > 2 years of follow up. Table S8: Associations between fruit juice intake and risk of overall and subsite-specific colorectal cancer for participants with > 2 years of follow up. Table S9: Age-adjusted associations between sweet beverage intake and risk of overall and subsite-specific colorectal cancer in the sample size of Model 2. Table S10: Associations between SSB intake and risk of overall and subsite-specific colorectal cancer not censoring for missing follow-up values. Table S11: Associations between ASB intake and risk of overall and subsite-specific colorectal cancer not censoring for missing follow-up values. Table S12: Associations between juice intake and risk of overall and subsite-specific colorectal cancer not censoring for missing follow-up values. Table S13: Associations between sweet beverage intake and risk of colorectal cancer by groups of baseline age, education level, and diabetes status. Table S14: Associations between sweet beverage intake and risk of overall and subsite-specific colorectal cancer adjusted for established risk factors.

## Data Availability

The dataset analyzed during the current study is not publicly available due to the sensitive nature of the data and we do not have permission from the participants to share data publicly. Access to the data can be requested through an application to the Norwegian Women and Cancer Study: https://uit.no/research/nowac_en/project?pid=824578.

## Declarations

The study has used data from the Cancer Registry of Norway. The interpretation and reporting of these data are the sole responsibility of the authors, and no endorsement by the Cancer Registry of Norway is intended nor should be inferred. Where authors are identified as personnel of the International Agency for Research on Cancer/World Health Organization, the authors alone are responsible for the views expressed in this article and they do not necessarily represent the decisions, policy or views of the International Agency for Research on Cancer/World Health Organization.

## Abbreviations

ASB: Artificially sweetened beverage
CIMP: CpG island methylator phenotype
CIN: Chromosomal instability
CRC: Colorectal cancer
DAG: Directed acyclic graph
FFQ: Food frequency questionnaire
HDI: Human development index
ICD-10: International Classification of Diseases 10th Revision
ICD-O-3: International Classification of Diseases for Oncology 3rd edition
MSI: Microsatellite instable
NHS: Nurses’ Health Study
NOWAC: Norwegian Women and Cancer
REK: Regional Committee for Medical and Health Research Ethics
SSB: Sugar-sweetened beverage
