# Supplementary material for: Sweet beverages and the risk of colorectal cancer: the Norwegian Women and Cancer Study
Source: BMC Cancer. 2025 Apr 1;25:592. doi: 10.1186/s12885-025-13835-4 (PMC11963648; doi:10.1186/s12885-025-13835-4)
Supplement: Supplementary file 1 — Supplementary Material 1: Figure S1: Directed acyclic graph for the association between SSB consumption and colorectal cancer incidence. Figure S2: Directed acyclic graph for the association between ASB consumption and colorectal cancer incidence. Figure S3: Directed acyclic graph for the association between juice consumption and colorectal cancer incidence. Table S1: Morphology codes (ICD-O-3) of adenocarcinomas for the cases in this study. Table S2: Cross-tabulation of SSB intake at baseline and follow-up. Table S3: Cross-tabulation of ASB intake at baseline and follow-up. Table S4: Cross-tabulation of juice intake at baseline and follow-up. Table S5: Associations between sweet beverage intake and risk of overall and subsite-specific colorectal cancer per 1 glass (250 ml) daily increase. Table S6: Associations between SSB intake and risk of overall and subsite-specific colorectal cancer for participants with > 2 years of follow up. Table S7: Associations between ASB intake and risk of overall and subsite-specific colorectal cancer for participants with > 2 years of follow up. Table S8: Associations between fruit juice intake and risk of overall and subsite-specific colorectal cancer for participants with > 2 years of follow up. Table S9: Age-adjusted associations between sweet beverage intake and risk of overall and subsite-specific colorectal cancer in the sample size of Model 2. Table S10: Associations between SSB intake and risk of overall and subsite-specific colorectal cancer not censoring for missing follow-up values. Table S11: Associations between ASB intake and risk of overall and subsite-specific colorectal cancer not censoring for missing follow-up values. Table S12: Associations between juice intake and risk of overall and subsite-specific colorectal cancer not censoring for missing follow-up values. Table S13: Associations between sweet beverage intake and risk of colorectal cancer by groups of baseline age, education level, and diabetes status. Table [file 12885_2025_13835_MOESM1_ESM.pdf]

Additional file 1

Figure S1: Directed acyclic graph for the association between SSB consumption and colorectal cancer incidence

Figure S2: Directed acyclic graph for the association between ASB consumption and colorectal cancer incidence

Figure S3: Directed acyclic graph for the association between juice consumption and colorectal cancer incidence

Table S1: Morphology codes (ICD-O-3) of adenocarcinomas for the cases in this study

Table S2: Cross-tabulation of SSB intake at baseline and follow-up

Table S3: Cross-tabulation of ASB intake at baseline and follow-up

Table S4: Cross-tabulation of juice intake at baseline and follow-up

Table S5: Associations between sweet beverage intake and risk of overall and subsite-specific colorectal cancer per 1 glass (250 ml) daily increase

Table S6: Associations between SSB intake and risk of overall and subsite-specific colorectal cancer for participants with > 2 years of follow up

Table S7: Associations between ASB intake and risk of overall and subsite-specific colorectal cancer for participants with > 2 years of follow up

Table S8: Associations between fruit juice intake and risk of overall and subsite-specific colorectal cancer for participants with > 2 years of follow up

Table S9: Age-adjusted associations between sweet beverage intake and risk of overall and subsite-specific colorectal cancer in the sample size of Model 2

Table S10: Associations between SSB intake and risk of overall and subsite-specific colorectal cancer not censoring for missing follow-up values

Table S11: Associations between ASB intake and risk of overall and subsite-specific colorectal cancer not censoring for missing follow-up values

Table S12: Associations between juice intake and risk of overall and subsite-specific colorectal cancer not censoring for missing follow-up values

Table S13: Associations between sweet beverage intake and risk of colorectal cancer by groups of baseline age, education level, and diabetes status

Table S14: Associations between sweet beverage intake and risk of overall and subsite-specific colorectal cancer adjusted for established risk factors

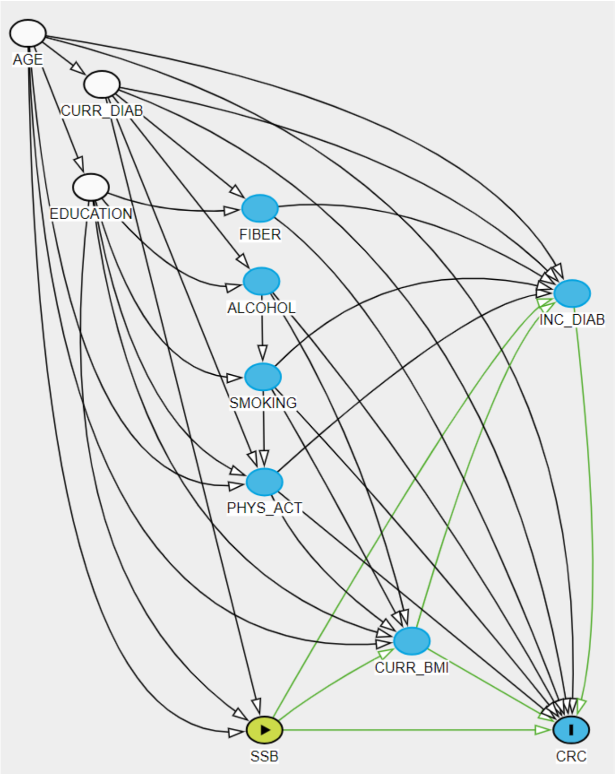

Figure S1. Directed acyclic graph for the association between SSB consumption and colorectal cancer incidence. Green node: exposure. White nodes: identified and adjusted confounders on the causal pathways. Blue node with I: outcome. Green paths: causal pathways. Black paths: closed paths. Abbreviations: SSB = sugar-sweetened beverage, CURR = current, DIAB = diabetes, INC = incident, PHYS_ACT = physical activity, BMI = body mass index, CRC = colorectal cancer

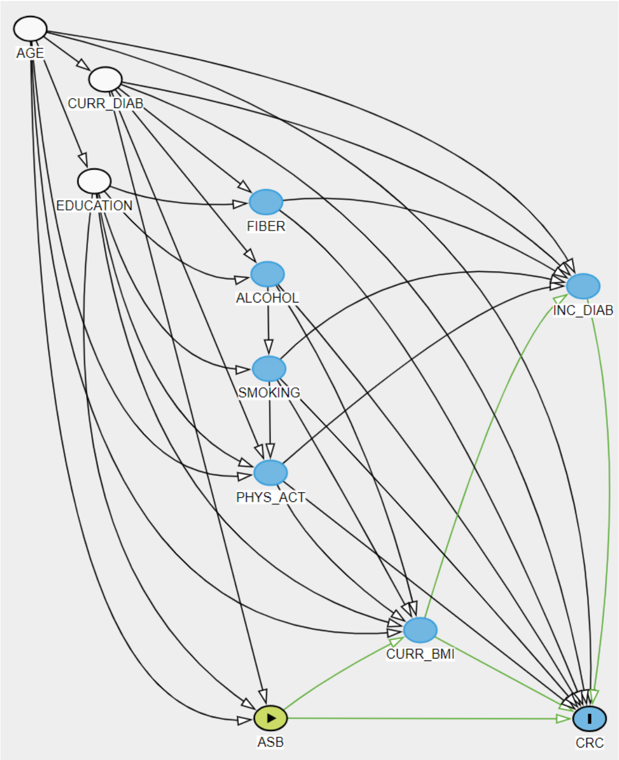

Figure S2. Directed acyclic graph for the association between ASB consumption and colorectal cancer incidence. Green node: exposure. White nodes: identified and adjusted confounders on the causal pathways. Blue node with I: outcome. Green paths: causal pathways. Black paths: closed paths. Abbreviations: ASB = artificially sweetened beverage, CURR = current, DIAB = diabetes, INC = incident, PHYS_ACT = physical activity, BMI = body mass index, CRC = colorectal cancer

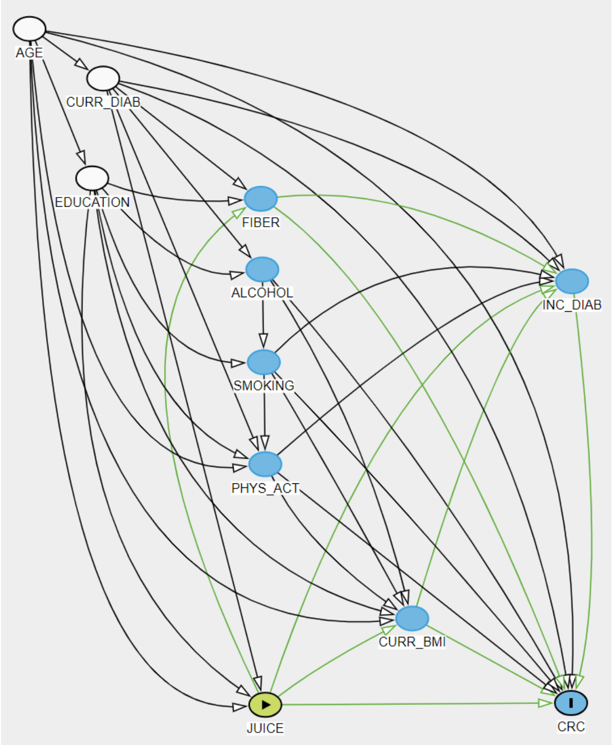

Figure S3. Directed acyclic graph for the association between juice consumption and colorectal cancer incidence. Green node: exposure. White nodes: identified and adjusted confounders on the causal pathways. Blue node with I: outcome. Green paths: causal pathways. Black paths: closed paths. Abbreviations: CURR = current, DIAB = diabetes, INC = incident, PHYS_ACT = physical activity, BMI = body mass index, CRC = colorectal cancer

Table S1. Morphology codes (ICD-O-3) of adenocarcinomas for the cases in this study

ADENOCARCINOMA, NOS	8140/3
ADENOCARCINOMA IN ADENOMATOUS POLYP	8210/3
TUBULAR ADENOCARCINOMA	8211/3
ADENOCARCINOMA WITH MIXED SUBTYPES	8255/3
PAPILLARY ADENOCARCINOMA, NOS	8260/3
ADENOCARCINOMA IN VILLOUS ADENOMA	8261/3
VILLOUS ADENOCARCINOMA	8262/3
ADENOCARCINOMA IN TUBULOVILLOUS ADENOMA	8263/3
MUCINOUS ADENOCARCINOMA	8480/3
MUCIN-PRODUCING ADENOCARCINOMA	8481/3
ADENOCARCINOMA WITH NEUROENDOCRINE DIFFERENC.	8574/3
MUCINOUS CYSTADENOCARCINOMA, NOS	8470/3

Table S2. Cross-tabulation of SSB intake at baseline and follow-up

SSB intake at baseline	SSB intake at follow-up			
	never/seldom	1-6 glasses/week	≥ 7 glasses/week	Total
never/seldom	51	4	0	55
1-6 glasses/week	23	13	1	37
≥ 7 glasses/week	3	3	2	8
Total	77	20	3	100

Table S3. Cross-tabulation of ASB intake at baseline and follow-up

ASB intake at baseline	ASB intake at follow-up			
	never/seldom	1-6 glasses/week	≥ 7 glasses/week	Total
never/seldom	55	7	1	63
1-6 glasses/week	12	12	3	27
≥ 7 glasses/week	2	4	4	10
Total	69	23	8	100

Table S4. Cross-tabulation of juice intake at baseline and follow-up

Juice intake at baseline	Juice intake at follow-up			
	never/seldom	1-6 glasses/week	≥ 7 glasses/week	Total
never/seldom	24	10	3	37
1-6 glasses/week	13	21	9	43
≥ 7 glasses/week	4	6	10	20
Total	41	37	22	100

Table S5. Associations between sweet beverage intake and risk of overall and subsite-specific colorectal cancer per 1 glass (250 ml) daily increase

Per 210 ml daily increase	Model 1				Model 2				
**SSB**	**n/cases**	**HR**	**95% CI**	**p-value**	**n/cases**	**HR**	**95% CI**	**p-value**	**p-het**
TOTAL CRC	62,549/714	1.02	0.86-1.21	0.796	60,259/687	1.00	0.84-1.20	0.997	
COLON CANCER	62,549/464	0.96	0.76-1.21	0.714	60,259/448	0.95	0.75-1.20	0.639	
PROXIMAL COLON CANCER	65,549/272	0.88	0.63-1.23	0.468	60,259/262	0.87	0.62-1.22	0.416	
DISTAL COLON CANCER	62,549/182	1.07	0.78-1.47	0.690	60,259/176	1.05	0.76-1.45	0.758	0.695[1]
RECTAL CANCER	62,549/250	1.13	0.87-1.46	0.360	60,259/239	1.09	0.83-1.44	0.522	0.798[2]
**ASB**									
TOTAL CRC	60,064/722	0.97	0.87-1.09	0.597	57,858/692	0.97	0.87-1.09	0.667	
COLON CANCER	60,064/479	0.99	0.87-1.14	0.920	57,858/463	0.99	0.86-1.14	0.871	
PROXIMAL COLON CANCER	60,064/278	1.04	0.88-1.24	0.612	57,858/268	1.04	0.87-1.24	0.677	
DISTAL COLON CANCER	60,064/192	0.94	0.75-1.18	0.594	57,858/186	0.95	0.75-1.19	0.631	0.184[1]
RECTAL CANCER	60,064/243	0.92	0.75-1.13	0.445	57,858/229	0.95	0.77-1.17	0.606	0.356[2]
**Juice**									
TOTAL CRC	68,089/822	0.88	0.77-1.00	0.050	65,391/791	0.90	0.79-1.03	0.112	
COLON CANCER	68,089/547	0.83	0.80-0.98	0.025	65,391/529	0.85	0.72-1.00	0.054	
PROXIMAL COLON CANCER	68,089/316	0.86	0.69-1.06	0.154	65,391/304	0.88	0.71-1.09	0.252	
DISTAL COLON CANCER	68,089/220	0.80	0.61-1.04	0.094	65,391/214	0.81	0.62-1.06	0.129	0.628[1]
RECTAL CANCER	68,089/275	0.98	0.79-1.20	0.823	65,391/262	1.00	0.81-1.24	0.985	0.231[2]

[1]p-value for heterogeneity between proximal and distal colon cancer
[2]p-value for heterogeneity between colon cancer and rectal cancer
Model 1: Adjusted for age
Model 2: Adjusted for age, education, and diabetes status at baseline
Abbreviations: SSB = sugar-sweetened beverage, n = number of observations, HR = hazard ratio, CI = confidence interval, CRC = colorectal cancer, ASB = artificially sweetened beverage

Table S6. Associations between SSB intake and risk of overall and subsite-specific colorectal cancer for participants with > 2 years of follow up

	Model 1					Model 2					
	**n/cases**	**HR**	**95% CI**	**p-value**	**p-trend**	**n/cases**	**HR**	**95% CI**	**p-value**	**p-trend**	**p-het**
**TOTAL CRC**	61,781/657					59,516/634					
never/seldom	41,987/474	1.00				40,405/457	1.00				
1-6 glasses/week	16,365/147	0.86	0.72-1.04	0.119		15,789/143	0.86	0.71-1.03	0.108		
≥ 7 glasses/week	3,429/36	1.12	0.79-1.57	0.530		3,322/34	1.06	0.74-1.50	0.758		
					0.537					0.394	
**COLON CANCER**	61,781/430					59,516/416					
never/seldom	41,987/305	1.00				40,405/295	1.00				
1-6 glasses/week	16,365/104	0.97	0.78-1.22	0.800		15,789/100	0.94	0.75-1.19	0.624		
≥ 7 glasses/week	3,429/21	1.04	0.67-1.62	0.867		3,322/21	1.03	0.66-1.61	0.897		
					0.953					0.812	
**PROXIMAL COLON CANCER**	61,781/258					59,516/248					
never/seldom	41,987/181	1.00				40,405/174	1.00				
1-6 glasses/week	16,365/66	1.10	0.83-1.46	0.515		15,789/63	1.07	0.80-1.43	0.652		
≥ 7 glasses/week	3,429/11	0.98	0.53-1.80	0.936		3,322/11	0.98	0.53-1.81	0.952		
					0.698					0.793	
**DISTAL COLON CANCER**	61,781/163					59,516/159					
never/seldom	41,987/116	1.00				40,405/113	1.00				
1-6 glasses/week	16,365/38	0.86	0.60-1.24	0.421		15,789/37	0.83	0.57-1.21	0.339		
≥ 7 glasses/week	3,429/9	1.07	0.54-2.11	0.847		3,322/9	1.03	0.52-2.05	0.925		
					0.688					0.577	0.107[1]
**RECTAL CANCER**	61,781/227					59,516/218					
never/seldom	41,987/169	1.00				40,405/162	1.00				
1-6 glasses/week	16,365/43	0.68	0.48-0.95	0.023		15,789/43	0.70	0.50-0.98	0.041		
≥ 7 glasses/week	3,429/15	1.24	0.73-2.12	0.426		3,322/13	1.10	0.62-1.95	0.738		
					0.338					0.264	0.569[2]

[1]p-value for heterogeneity between proximal and distal colon cancer
[2]p-value for heterogeneity between colon cancer and rectal cancer
Model 1: Adjusted for age
Model 2: Adjusted for age, education, and diabetes status at baseline
Abbreviations: n = number of observations, HR = hazard ratio, CI = confidence interval, CRC = colorectal cancer
1 glass corresponds to 210 ml

Table S7. Associations between ASB intake and risk of overall and subsite-specific colorectal cancer for participants with > 2 years of follow up

	Model 1					Model 2					
	n/cases	HR	95% CI	p-value	p-trend	n/cases	HR	95% CI	p-value	p-trend	p-Het
**TOTAL CRC**	59,326/670					57,148/644					
never/seldom	39,584/441	1.00				38,132/427	1.00				
1-6 glasses/week	14,185/177	1.18	0.99-1.40	0.068		13,664/166	1.13	0.95-1.36	0.170		
≥ 7 glasses/week	5,557/52	0.92	0.69-1.23	0.578		5,352/51	0.93	0.70-1.25	0.634		
					0.604					0.735	
**COLON CANCER**	59,326/446					57,148/432					
never/seldom	39,584/285	1.00				38,132/278	1.00				
1-6 glasses/week	14,185/125	1.30	1.05-1.60	0.015		13,664/119	1.25	1.00-1.55	0.046		
≥ 7 glasses/week	5,557/36	1.00	0.71-1.41	0.996		5,352/35	0.98	0.69-1.39	0.901		
					0.212					0.357	
**PROXIMAL COLON CANCER**	59,326/265					57,148/255					
never/seldom	39,584/171	1.00				38,132/166	1.00				
1-6 glasses/week	14,185/72	1.27	0.97-1.68	0.086		13,664/68	1.23	0.92-1.63	0.157		
≥ 7 glasses/week	5,557/22	1.05	0.67-1.63	0.839		5,352/21	1.02	0.65-1.61	0.935		
					0.296					0.431	
**DISTAL COLON CANCER**	59,326/173					57,148/169					
never/seldom	39,584/108	1.00				38,132/106	1.00				
1-6 glasses/week	14,185/51	1.35	0.97-1.89	0.076		13,664/49	1.30	0.92-1.82	0.135		
≥ 7 glasses/week	5,557/14	0.98	0.56-1.72	0.955		5,352/14	0.98	0.56-1.73	0.956		
					0.385					0.474	0.156[1]
**RECTAL CANCER**	59,326/224					57,148/212					
never/seldom	39,584/156	1.00				38,132/149	1.00				
1-6 glasses/week	14,185/52	0.96	0.70-1.31	0.799		13,664/47	0.93	0.67-1.29	0.652		
≥ 7 glasses/week	5,557/16	0.78	0.47-1.31	0.352		5,352/16	0.85	0.50-1.42	0.527		
					0.392					0.469	0.089[2]

[1]p-value for heterogeneity between proximal and distal colon cancer
[2]p-value for heterogeneity between colon cancer and rectal cancer
Model 1: Adjusted for age
Model 2: Adjusted for age, education, and diabetes status at baseline
Abbreviations: n = number of observations, HR = hazard ratio, CI = confidence interval, CRC = colorectal cancer
1 glass corresponds to 210 ml

Table S8. Associations between fruit juice intake and risk of overall and subsite-specific colorectal cancer for participants with > 2 years of follow up

	Model 1					Model 2					
	**n/cases**	**HR**	**95% CI**	**p-value**	**p-trend**	**n/cases**	**HR**	**95% CI**	**p-value**	**p-trend**	**p-het**
**TOTAL CRC**	67,256/761					64,596/735					
never/seldom	27,353/340	1.00				26,252/323	1.00				
1-6 glasses/week	25,748/273	0.83	0.71-0.98	0.025		24,723/267	0.86	0.73-1.02	0.078		
≥ 7 glasses/week	14,155/148	0.80	0.66-0.97	0.026		13,621/145	0.84	0.69-1.02	0.074		
					0.012					0.046	
**COLON CANCER**	67,256/506					64,596/491					
never/seldom	27,353/229	1.00				26,252/219	1.00				
1-6 glasses/week	25,748/191	0.87	0.72-1.06	0.161		24,723/186	0.89	0.73-1.09	0.262		
≥ 7 glasses/week	14,155/86	0.69	0.54-0.89	0.004		13,621/86	0.73	0.57-0.94	0.015		
					0.004					0.016	
**PROXIMAL COLON CANCER**	67,256/299					64,596/288					
never/seldom	27,353/133	1.00				26,252/126	1.00				
1-6 glasses/week	25,748/119	0.95	0.74-1.21	0.673		24,723/115	0.97	0.75-1.25	0.803		
≥ 7 glasses/week	14,155/47	0.65	0.47-0.91	0.012		13,621/47	0.69	0.49-0.96	0.030		
					0.020					0.048	
**DISTAL COLON CANCER**	67,256/198					64,596/194					
never/seldom	27,353/92	1.00				26,252/89	1.00				
1-6 glasses/week	25,748/69	0.77	0.56-1.05	0.098		24,723/68	0.79	0.58-1.09	0.151		
≥ 7 glasses/week	14,155/37	0.75	0.51-1.09	0.124		13,621/37	0.78	0.54-1.16	0.223		
					0.076					0.152	0.812[1]
**RECTAL CANCER**	67,256/255					64,596/244					
never/seldom	27,353/111	1.00				26,252/104	1.00				
1-6 glasses/week	25,748/82	0.76	0.57-1.01	0.056		24,723/81	0.80	0.60-1.07	0.140		
≥ 7 glasses/week	14,155/62	1.03	0.75-1.40	0.856		13,621/59	1.05	0.76-1.44	0.774		
					0.825					0.975	0.356[2]

[1]p-value for heterogeneity between proximal and distal colon cancer
[2]p-value for heterogeneity between colon cancer and rectal cancer
Model 1: Adjusted for age
Model 2: Adjusted for age, education, and diabetes status at baseline
Abbreviations: n = number of observations, HR = hazard ratio, CI = confidence interval, CRC = colorectal cancer
1 glass corresponds to 210 ml

Table S9. Age-adjusted associations between sweet beverage intake and risk of overall and subsite-specific colorectal cancer in the sample size of Model 2

	SSB					ASB					Juice				
	**n/cases**	**HR**	**95% CI**	**p-value**	**p-trend**	**n/cases**	**HR**	**95% CI**	**p-value**	**p-trend**	**n/cases**	**HR**	**95% CI**	**p-value**	**p-trend**
**TOTAL CRC**	60,259/687					57,858/692					65,391/791				
never/seldom	40,865/486	1.00				38,596/457	1.00				26,597/345	1.00			
1-6 glasses/week	16,012/160	0.88	0.74-1.06	0.172		13,837/180	1.14	0.96-1.36	0.135		25,031/295	0.88	0.75-1.03	0.109	
≥ 7 glasses/week	3,382/41	1.18	0.86-1.62	0.313		5,425/55	0.93	0.71-1.23	0.596		13,763/151	0.81	0.67-0.98	0.027	
					0.825					0.717					0.019
**COLON CANCER**	60,259/448					57,858/463					65,391/529				
never/seldom	40,865/313	1.00				38,596/295	1.00				26,597/233	1.00			
1-6 glasses/week	16,012/111	0.97	0.78-1.21	0.787		13,837/129	1.28	1.04-1.57	0.021		25,031/204	0.91	0.75-1.09	0.305	
≥ 7 glasses/week	3,382/24	1.10	0.72-1.66	0.667		5,425/39	1.03	0.74-1.44	0.865		13,763/92	0.73	0.57-0.93	0.010	
					0.915					0.189					0.011
**PROXIMAL COLON CANCER**	60,259/262					57,858/268					65,391/304				
never/seldom	40,865/182	1.00				38,596/172	1.00				26,597/131	1.00			
1-6 glasses/week	16,012/68	1.09	0.82-1.44	0.566		13,837/72	1.25	0.95-1.65	0.108		25,031/122	0.98	0.77-1.25	0.869	
≥ 7 glasses/week	3,382/12	1.01	0.56-1.81	0.984		5,425/24	1.12	0.73-1.72	0.593		13,763/51	0.72	0.51-0.99	0.044	
					0.691					0.219					0.070
**DISTAL COLON CANCER**	60,259/176					57,858/186					65,391/214				
never/seldom	40,865/122	1.00				38,596/116	1.00				26,597/97	1.00			
1-6 glasses/week	16,012/43	0.89	0.62-1.26	0.585		13,837/55	1.34	0.97-1.84	0.076		25,031/78	0.82	0.61-1.10	0.180	
≥ 7 glasses/week	3,382/11	1.18	0.63-2.19	0.605		5,425/15	0.96	0.56-1.65	0.891		13,763/39	0.74	0.51-1.07	0.111	
					0.932					0.422					0.081
p-heterogeneity[1]					0.155					0.112					0.572
**RECTAL CANCER**	60,259/239					57,858/229					65,391/262				
never/seldom	40,865/173	1.00				38,596/162	1.00				26,597/112	1.00			
1-6 glasses/week	16,012/49	0.73	0.53-1.00	0.053		13,837/51	0.90	0.65-1.23	0.502		25,031/91	0.83	0.63-1.09	0.181	
≥ 7 glasses/week	3,382/17	1.32	0.80-2.18	0.279		5,425/16	0.74	0.45-1.25	0.261		13,763/59	0.97	0.71-1.33	0.841	
					0.606					0.220					0.644
p-heterogeneity[2]					0.689					0.022					0.502

[1]p-value for heterogeneity between proximal and distal colon cancer
[2]p-value for heterogeneity between colon cancer and rectal cancer
Abbreviations: SSB = sugar-sweetened beverage, ASB = artificially sweetened beverage, n = number of observations, HR = hazard ratio, CI = confidence interval, CRC = colorectal cancer
1 glass corresponds to 210 ml

Table S10. Associations between SSB intake and risk of overall and subsite-specific colorectal cancer not censoring for missing follow-up values

	Model 1					Model 2					
	**n/cases**	**HR**	**95% CI**	**p-value**	**p-trend**	**n/cases**	**HR**	**95% CI**	**p-value**	**p-trend**	**p-het**
**TOTAL CRC**	62,549/977					60,259/940					
never/seldom	42,464/648	1.00				40,865/624	1.00				
1-6 glasses/week	16,595/257	0.92	0.79-1.06	0.248		16,012/248	0.91	0.78-1.05	0.205		
≥ 7 glasses/week	3,490/72	1.24	0.97-1.58	0.085		3,382/68	1.18	0.92-1.52	0.197		
					0.646					0.917	
**COLON CANCER**	62,549/646					60,259/624					
never/seldom	42,464/423	1.00				40,865/409	1.00				
1-6 glasses/week	16,595/182	1.01	0.85-1.21	0.891		16,012/175	0.98	0.82-1.17	0.839		
≥ 7 glasses/week	3,490/41	1.10	0.80-1.51	0.567		3,382/40	1.06	0.76-1.47	0.730		
					0.635					0.913	
**PROXIMAL COLON CANCER**	62,549/384					60,259/370					
never/seldom	42,464/253	1.00				40,865/244	1.00				
1-6 glasses/week	16,595/108	1.04	0.83-1.30	0.732		16,012/104	1.02	0.81-1.29	0.857		
≥ 7 glasses/week	3,490/23	1.07	0.70-1.63	0.770		3,382/22	1.03	0.66-1.59	0.908		
					0.678					0.848	
**DISTAL COLON CANCER**	62,549/249					60,259/241					
never/seldom	42,464/160	1.00				40,865/155	1.00				
1-6 glasses/week	16,595/73	1.02	0.77-1.35	0.895		16,012/70	0.97	0.73-1.29	0.825		
≥ 7 glasses/week	3,490/16	1.08	0.64-1.80	0.778		3,382/16	1.04	0.62-1.74	0.896		
					0.784					0.963	0.273[1]
**RECTAL CANCER**	62,549/331					60,259/316					
never/seldom	42,464/225	1.00				40,865/215	1.00				
1-6 glasses/week	16,595/75	0.75	0.58-0.97	0.030		16,012/73	0.77	0.59-1.01	0.056		
≥ 7 glasses/week	3,490/31	1.49	1.02-2.17	0.038		3,382/28	1.41	0.95-2.10	0.089		
					0.899					0.977	0.921[2]

[1]p-value for heterogeneity between proximal and distal colon cancer
[2]p-value for heterogeneity between colon cancer and rectal cancer
Model 1: Adjusted for age
Model 2: Adjusted for age, education, and diabetes status at baseline
Abbreviations: n = number of observations, HR = hazard ratio, CI = confidence interval, CRC = colorectal cancer
1 glass corresponds to 210 ml

Table S11. Associations between ASB intake and risk of overall and subsite-specific colorectal cancer not censoring for missing follow-up values

	Model 1					Model 2					
	**n/cases**	**HR**	**95% CI**	**p-value**	**p-trend**	**n/cases**	**HR**	**95% CI**	**p-value**	**p-trend**	**p-het**
**TOTAL CRC**	60,064/958					57,858/920					
never/seldom	40,064/617	1.00				38,596/597	1.00				
1-6 glasses/week	14,367/263	1.18	1.02-1.36	0.027		13,837/247	1.13	0.97-1.31	0.106		
≥ 7 glasses/week	5,633/78	0.91	0.72-1.15	0.430		5,425/76	0.90	0.71-1.15	0.406		
					0.608					0.868	
**COLON CANCER**	60,064/642					57,858/620					
never/seldom	40,064/406	1.00				38,596/395	1.00				
1-6 glasses/week	14,367/183	1.25	1.05-1.49	0.011		13,837/173	1.20	1.00-1.44	0.049		
≥ 7 glasses/week	5,633/53	0.95	0.71-1.26	0.713		5,425/52	0.94	0.70-1.25	0.658		
					0.316					0.520	
**PROXIMAL COLON CANCER**	60,064/383					57,858/369					
never/seldom	40,064/241	1.00				38,596/235	1.00				
1-6 glasses/week	14,367/109	1.28	1.02-1.61	0.032		13,837/102	1.22	0.96-1.54	0.103		
≥ 7 glasses/week	5,633/33	1.02	0.71-1.46	0.931		5,425/32	0.99	0.69-1.44	0.979		
					0.250					0.427	
**DISTAL COLON CANCER**	60,064/248					57,858/240					
never/seldom	40,064/157	1.00				28,596/152	1.00				
1-6 glasses/week	14,367/72	1.24	0.94-1.64	0.128		13,837/69	1.20	0.90-1.60	0.204		
≥ 7 glasses/week	5,633/19	0.85	0.53-1.37	0.507		5,425/19	0.86	0.53-1.39	0.541		
					0.792					0.868	0.127[1]
**RECTAL CANCER**	60,064/316					57,858/300					
never/seldom	40,064/211	1.00				38,596/202	1.00				
1-6 glasses/week	14,367/80	1.03	0.80-1.34	0.810		13,837/74	1.00	0.76-1.31	1.000		
≥ 7 glasses/week	5,633/25	0.84	0.55-1.27	0.399		5,425/24	0.84	0.55-1.28	0.416		
					0.594					0.527	0.159[2]

[1]p-value for heterogeneity between proximal and distal colon cancer
[2]p-value for heterogeneity between colon cancer and rectal cancer
Model 1: Adjusted for age
Model 2: Adjusted for age, education, and diabetes status at baseline
Abbreviations: n = number of observations, HR = hazard ratio, CI = confidence interval, CRC = colorectal cancer
1 glass corresponds to 210 ml

Table S12. Associations between juice intake and risk of overall and subsite-specific colorectal cancer not censoring for missing follow-up values

	Model 1					Model 2					
	**n/cases**	**HR**	**95% CI**	**p-value**	**p-trend**	**n/cases**	**HR**	**95% CI**	**p-value**	**p-trend**	**p-het**
**TOTAL CRC**	68,089/1,070					65,391/1,031					
never/seldom	27,709/463	1.00				26,597/443	1.00				
1-6 glasses/week	26,075/413	0.91	0.79-1.04	0.152		25,031/400	0.93	0.81-1.06	0.290		
≥ 7 glasses/week	14,305/194	0.79	0.67-0.94	0.008		13,763/188	0.82	0.69-0.97	0.023		
					0.007					0.023	
**COLON CANCER**	68,089/721					65,391/697					
never/seldom	27,709/313	1.00				26,597/301	1.00				
1-6 glasses/week	26,075/287	0.94	0.80-1.10	0.435		25,031/277	0.95	0.81-1.12	0.553		
≥ 7 glasses/week	14,305/121	0.74	0.60-0.91	0.004		13,763/119	0.77	0.62-0.95	0.014		
					0.007					0.022	
**PROXIMAL COLON CANCER**	68,089/426					65,391/409					
never/seldom	27,709/184	1.00				26,597/176	1.00				
1-6 glasses/week	26,075/172	0.97	0.79-1.19	0.767		25,031/164	0.97	0.78-1.20	0.784		
≥ 7 glasses/week	14,305/70	0.73	0.55-0.96	0.023		13,763/69	0.75	0.57-1.00	0.046		
					0.038					0.069	
**DISTAL COLON CANCER**	68,089/280					65,391/273					
never/seldom	27,709/124	1.00				26,597/120	1.00				
1-6 glasses/week	26,075/108	0.87	0.68-1.13	0.309		25,031/106	0.90	0.69-1.17	0.441		
≥ 7 glasses/week	14,305/48	0.73	0.53-1.02	0.069		13,763/47	0.77	0.55-1.08	0.126		
					0.062					0.124	0.709[1]
**RECTAL CANCER**	68,089/349					65,391/334					
never/seldom	27,709/150	1.00				26,597/142	1.00				
1-6 glasses/week	26,075/126	0.84	0.67-1.07	0.163		25,031/123	0.88	0.69-1.13	0.314		
≥ 7 glasses/week	14,305/73	0.92	0.70-1.22	0.569		13,763/69	0.93	0.70-1.24	0.625		
					0.409					0.507	0.705[2]

[1]p-value for heterogeneity between proximal and distal colon cancer
[2]p-value for heterogeneity between colon cancer and rectal cancer
Model 1: Adjusted for age
Model 2: Adjusted for age, education, and diabetes status at baseline
Abbreviations: n = number of observations, HR = hazard ratio, CI = confidence interval, CRC = colorectal cancer
1 glass corresponds to 210 ml

Table S13. Associations between sweet beverage intake and risk of colorectal cancer by groups of baseline age, education level, and diabetes status

	SSB					ASB					Juice				
	n/cases	HR	95% CI	p-value	p-trend	n/cases	HR	95% CI	p-value	p-trend	n/cases	HR	95% CI	p-value	p-trend
**41-50 y[1]**	29,401/237					28,137/239					31,046/262				
never/seldom	18,902/153	1.00				18,002/154	1.00				11,952/107	1.00			
1-6 glasses/week	8,635/65	1.00	0.74-1.34	0.975		7,219/62	0.99	0.74-1.34	0.958		12,434/112	0.97	0.75-1.27	0.849	
≥ 7 glasses/week	1,864/19	1.50	0.93-2.43	0.100		2,916/23	0.93	0.60-1.45	0.757		6,660/43	0.67	0.47-0.96	0.028	
					0.278					0.789					0.046
**51-61 y[1]**	34,492/493					33,256/500					38,301/577				
never/seldom	24,462/361	1.00				22,947/333	1.00				16,283/257	1.00			
1-6 glasses/week	8,314/110	0.85	0.68-1.05	0.131		7,462/131	1.22	0.99-1.49	0.059		14,133/201	0.85	0.71-1.02	0.083	
≥ 7 glasses/week	1,716/22	0.88	0.67-1.35	0.547		2,847/36	0.91	0.65-1.29	0.602		7,885/119	0.91	0.73-1.13	0.399	
					0.160					0.552					0.247
**< 10 y EDUCATION[2]**	11,316/157					10,642/145					12,244/167				
never/seldom	6,836/100	1.00				6,549/85	1.00				5,645/71	1.00			
1-6 glasses/week	3,538/46	0.99	0.70-1.41	0.964		2,923/47	1.27	0.89-1.82	0.193		4,559/78	1.35	0.98-1.86	0.069	
≥ 7 glasses/week	942/11	0.99	0.53-1.86	0.981		1,170/13	0.93	0.52-1.68	0.822		2,040/18	0.67	0.40-1.12	0.127	
					0.966					0.644					0.544
**10-12y EDUCATION[2]**	21,315/230					20,461/235					23,240/281				
never/seldom	13,969/164	1.00				13,113/155	1.00				9,881/131	1.00			
1-6 glasses/week	6,046/49	0.71	0.51-0.97	0.034		5,231/60	1.00	0.74-1.35	0.982		8,791/90	0.73	0.56-0.96	0.024	
≥ 7 glasses/week	1,300/17	1.26	0.76-2.07	0.376		2,117/20	0.85	0.53-1.36	0.495		4,568/60	0.93	0.68-1.26	0.617	
					0.458					0.601					0.320
**> 12y EDUCATION[2]**	27,628/300					26,755/312					29,907/343				
never/seldom	20,060/222	1.00				18,934/217	1.00				11,071/143	1.00			
1-6 glasses/week	6,428/65	0.95	0.71-1.25	0.694		5,683/73	1.18	0.91-1.54	0.218		11,681/127	0.82	0.65-1.04	0.104	
≥ 7 glasses/week	1,140/13	1.16	0.66-2.03	0.610		2,138/22	0.99	0.64-1.54	0.970		7,155/73	0.76	0.57-1.01	0.058	
					0.963					0.524					0.043
**NO DIABETES DIAGNOSIS[3]**	59,447/678					56,709/678					64,401/778				
never/seldom	40,111/479	1.00				38,233/455	1.00				25,965/338	1.00			

1-6 glasses/week	15,964/159	0.86	0.72-1.04	0.116		13,359/172	1.12	0.94-1.36	0.208		24,776/289	0.87	0.75-1.02	0.088	
≥ 7 glasses/week	3,372/40	1.12	0.81-1.55	0.497		5,117/51	0.91	0.68-1.21	0.512		13,660/151	0.82	0.67-0.99	0.038	
					0.571					0.875					0.025

[1]Adjusted for age, education, and diabetes status at baseline
[2]Adjusted for age and diabetes status at baseline
[3]Adjusted for age and education
Abbreviations: SSB = sugar-sweetened beverage, ASB = artificially sweetened beverage, n = number of observations, HR = hazard ratio, CI = confidence interval, CRC = colorectal cancer
1 glass corresponds to 210 ml

Table S14. Associations between sweet beverage intake and risk of overall and subsite-specific colorectal cancer adjusted for established risk factors

	SSB					ASB					Juice				
	n/cases	HR	95% CI	p-value	p-trend	n/cases	HR	95% CI	p-value	p-trend	n/cases	HR	95% CI	p-value	p-trend
**TOTAL CRC**	54,888/625					52,889/634					59,169/719				
never/seldom	37,466/448	1.00				35,427/423	1.00				24,094/316	1.00			
1-6 glasses/week	14,419/139	0.85	0.70-1.03	0.102		12,554/161	1.09	0.91-1.31	0.363		22,674/268	0.90	0.76-1.06	0.200	
≥ 7 glasses/week	3,003/38	1.17	0.84-1.65	0.350		4,908/50	0.88	0.65-1.18	0.390		12,401/135	0.81	0.66-1.00	0.049	
					0.680					0.837					0.041
**COLON CANCER**	54,888/410					52,889/424					59,169/480				
never/seldom	37,466/289	1.00				35,427/271	1.00				24,094/214	1.00			
1-6 glasses/week	14,419/97	0.89	0.71-1.14	0.369		12,554/117	1.21	0.97-1.50	0.093		22,674/184	0.92	0.75-1.12	0.392	
≥ 7 glasses/week	3,003/24	1.10	0.72-1.68	0.662		4,908/36	0.94	0.66-1.34	0.753		12,401/82	0.74	0.57-0.96	0.022	
					0.784					0.558					0.026
**PROXIMAL COLON CANCER**	54,888/243					52,889/249					59,169/283				
never/seldom	37,466/170	1.00				35,427/160	1.00				24,094/124	1.00			
1-6 glasses/week	14,419/61	1.03	0.78-1.39	0.861		12,554/66	1.19	0.89-1.59	0.236		22,674/111	0.98	0.75-1.30	0.859	
≥ 7 glasses/week	3,003/12	0.99	0.55-1.79	0.980		4,908/23	1.06	0.68-1.65	0.802		12,401/48	0.75	0.54-1.06	0.101	
					0.923					0.439					0.138
**DISTAL COLON CANCER**	54,888/158					52,889/166					59,169/187				
never/seldom	37,466/111	1.00				35,427/104	1.00				24,094/86	1.00			
1-6 glasses/week	14,419/36	0.79	0.54-1.16	0.236		12,554/49	1.27	0.90-1.79	0.173		22,674/69	0.83	0.60-1.14	0.257	
≥ 7 glasses/week	3,003/11	1.21	0.64-2.28	0.550		4,908/13	0.86	0.48-1.54	0.615		12,401/32	0.71	0.47-1.08	0.107	
					0.740					0.770					0.087
p-heterogeneity[1]					0.106					0.100					0.503
**RECTAL CANCER**	54,888/215					52,889/210					59,169/239				
never/seldom	37,466/159	1.00				35,427/152	1.00				24,094/102	1.00			
1-6 glasses/week	14,419/42	0.76	0.53-1.07	0.118		12,554/44	0.87	0.62-1.22	0.420		22,674/84	0.86	0.64-1.15	0.312	
≥ 7 glasses/week	3,003/14	1.33	0.76-2.33	0.311		4,908/14	0.75	0.43-1.30	0.308		12,401/53	0.96	0.69-1.35	0.835	
					0.743					0.225					0.692
p-heterogeneity[2]					0.977					0.104					0.430

[1]p-value for heterogeneity between proximal and distal colon cancer
[2]p-value for heterogeneity between colon cancer and rectal cancer
Analyses adjusted for age, education, diabetes status, smoking status, physical activity level, consumption of red and processed meat, alcohol, fiber and calcium at baseline
Abbreviations: SSB = sugar-sweetened beverage, ASB = artificially sweetened beverage, n = number of observations, HR = hazard ratio, CI = confidence interval, CRC = colorectal cancer
1 glass corresponds to 210 ml
